# Age-Related Subgingival Colonization of *Aggregatibacter actinomycetemcomitans*, *Porphyromonas gingivalis* and *Parvimonas micra*—A Pragmatic Microbiological Retrospective Report

**DOI:** 10.3390/microorganisms11061434

**Published:** 2023-05-29

**Authors:** Rolf Claesson, Anders Johansson, Georgios N. Belibasakis

**Affiliations:** 1 Department of Odontology, Umeå University, 901 87 Umeå, Sweden; anders.p.johansson@umu.se; 2 Division of Oral Diseases, Department of Dental Medicine, Karolinska Institutet, 14152 Huddinge, Sweden; george.belibasakis@ki.se

**Keywords:** periodontitis, age-related, *Aggregatibacter actinomycetemcomitans*, *Porphyromonas gingivalis*, *Parvimonas micra*

## Abstract

The aim of this study was to compare data about the prevalence and proportions of the bacterial species *Aggregatibacter actinomycetemcomitans*, *Porphyromonas gingivalis*, and *Parvimonas micra* in periodontitis pocket samples collected from young, <35 years, and old, >35-year-old patients, YP and OP, respectively. The results from the analyses of a total of 3447 subgingival plaque samples analyzed for clinical diagnosis purposes by cultivation regarding the proportions of these species were collected from a database and elucidated. The prevalence of A. *actinomycetemcomitans* was found to be more than twice as high (OR = 2.96, 95% CI; 2.50–3.50) in samples from the younger (42.2%) than the older group (20.4%) (*p* < 0.001). The prevalence of *P. micra* was significantly lower in samples from the younger age group (OR = 0.43, 95%) (*p* < 0.001), whereas *P. gingivalis* was similarly distributed (OR = 0.78, 95%) in the two age groups (*p* = 0.006). A similar pattern was noticed for *A. actinomycetemcomitans* and *P. gingivalis* when high proportions (>50%) of the samples of these bacterial species were elucidated. In contrast, the proportion of samples containing >50% with *P. micra* was lower compared with the two other bacterial species. Furthermore, it was noted that the proportion of samples from old patients containing *A. actinomycetemcomitans* in combination with *P. micra* was almost three times higher than in samples when *P. micra* was replaced by *P. gingivalis*. In conclusion, *A***.** *actinomycetemcomitans* showed an increased presence and proportion in samples from young patients compared with the old patients, while *P. gingivalis* was similarly distributed in the two age groups. *P. micra* showed an increased presence and proportion in samples from old patients compared with the young patients.

## 1. Introduction

Periodontitis is a multifactorial disease characterized by microbial-induced inflammatory destruction of the periodontal (tooth-supporting) tissues [1]. Oral hygiene status, genetic factors, and also physiological factors, such as gradual aging, contribute to the risk of the initiation and progression of the disease [2]. Several bacterial species, organized within biofilm communities that attach and grow on the tooth surface, are considered the major instigating factor of the disease [3,4,5]. While molecular and high-throughput methods have enhanced our appreciation of the vast diversity of microorganisms present in biofilms, a handful of those have been more extensively studied than others, mainly due to the fact that they are able to grow under laboratory conditions [6,7]. Among these are the gram-negative capnophilic rod *Aggregatibacter actinomycetemcomitans* [8] and the anaerobic rod *Porphyromonas gingivalis* [9]. The gram-positive anaerobic coccus *Parvimonas micra* (earlier *Peptostreptococcus micros* and *Micromonas micra*) has also been studied [10]. While *A. actinomycetemcomitans* is associated with aggressive forms of the periodontitis (grade C) affecting adolescents and young adults, *P. gingivalis* and *P. micra* are mainly associated with chronic, slow progressing forms of the disease that occur later in life [11].

The association of these bacterial species to disease may be, at least partly, due to a carriage of different virulence factors [2]. The leukotoxin of *A. actinomycetemcomitans* activates and kills immune cells by different mechanisms [12]. Individuals carrying the JP2 genotype of the bacterium, which produces increased levels of the leukotoxin, are at an increased risk of periodontitis [13,14]. Recently *A. actinomycetemcomitans* carrying the *cageE* gene was found to produce increased levels of this toxin [15]. *P. gingivalis* produces gingipains, which dysregulate a number of normally tightly controlled pathways [16]. *P. micra* activates those gingipains and also produces high amounts of hydrogen sulfide [17,18].

Rising age is an important host-related factor that influences the microbial ecology of the oral cavity. Subsequent changes in the oral microbial community over time may become detrimental for the oral and systemic health of an older (aging) individual [19,20,21].

When studying factors associated with the initiation and progression of periodontitis, the clinical parameters collected from the patients are crucial factors. However, when we recently pooled together 15 years (2000–2014) of long data from our database to investigate the presence of *A. actinomycetemcomitans* in dental plaque collected for routine clinical laboratory analysis, the clinical diagnosis of the patients was not homogeneously reported in the patient information attached to the referral to the laboratory for microbiological diagnostics. Thus, the classification of the patients was digitomized only and was based on the old definition of early onset periodontitis, which distinguished patients ≤35 years versus those >35 years of age [22].

That study showed an increased prevalence and proportion of *A. actinomycetemcomitans* in samples from patients <35 years compared to those of the in the samples from patients >35 years. Our hypothesis is that the presence of *A. actinomycetemcomitans* bacteria is gradually replaced by the obligate anaerobic bacterial species as the patient ages, and the microecology of the periodontal is modified to anoxic conditions.

Therefore, in this report, we aimed to compare and summarize clinical microbiological registry data on the presence of the capnophilic *A. actinomycetemcomitans* and of the obligate anaerobes *P. gingivalis* and *P. micra* in subgingival plaque samples sent by external referral for routine diagnostic analysis. The clinical diagnosis of the patients was not considered, although the data was age-stratified in a binary manner (≤35 years and >35 years).

## 2. Materials and Methods

### 2.1. Study Population and Collections

The study was performed at the Dental School, Umeå University, Sweden, and includes samples which were received from the undergraduate or/and specialist clinics of the dental school and from external clinics. Collected samples were microbiologically analyzed at the Clinical Oral Microbiology laboratory of the Dental School for periodontal routine diagnostic purposes over a period of 15 years. We underline that this study is a summary of data obtained from analyses of clinical samples sent to the clinical laboratory for identification and characterization. No sample has been originally taken for research purposes, and the data cannot be traced to any of the sampled individuals. In addition, no clinical data from the patients were included. From the database results from all analyzed subgingival plaque samples, a total of 3447, collected from 1505 unique patients (range 9–92 years), were used. Among these, 1087 samples were collected from patients ≤ 35 years, young patients (YP, n = 425 patients), whereas 2360 samples were collected from patients >35 years, old patients (OP, n = 1082 patients). Based on these data, this study was carried out according to the aim.

### 2.2. Cultivation and Quantification of Aggregatibacter actinomycetemcomitans, Porphyromonas gingivalis, and Parvimonas micra

The samples were routinely produced by using paper points and transported in a viable anaerobic medium (VMGAIII) to the clinical laboratory and handled as described previously [23]. Briefly, samples were serially diluted and spread on blood agar plates containing Columbia agar base (CBA), 5% defibrinated horse blood, 5 mg hemin/L, and 10 mg Vitamin K/L), followed by a seven-day incubation under anaerobic conditions (10% H_2_, 5% CO_2_, 85% N_2_). The colonies of *P. gingivalis* and *P. micra* colonies were macroscopically identified as being black pigmented and showing a positive BAPNA test, while *P. micra* colonies were identified as having a white smooth or rough morphology, surrounded by a halo [6,24]. For the detection of *A. actinomycetemomitans*, the sample were spread on trypticase–bacitracin–vancomycin (TBV) plates and incubated for three days under aerobic condition in the presence of 5% CO_2_, following macroscopic identification. The colonies of *A. actinomycetemcomitans* were identified as having a starlike structure and showing a positive catalase test [25]. Since usage of paper points does not provide a sample size with an internal standard, total viable count (TVC) was used. This allowed normalization of the results of the analyses between the samples. This parameter was also used for calculation of the proportions (%) of the bacterial species.

### 2.3. Comparison of Proportions and Concentrations of A. actinomycetemcomitans in Periodontal Pockets Samples

Since the proportion and concentration of *A. actinomycetemcomitans* are different parameters, in this report, we presented these parameters in relation to each other. The concentration values of the bacterium of each sample were like the proportion values collected from the database.

### 2.4. Statistical Analysis

Chi-square tests were used to identify significant differences in the distribution of the individuals and samples positive for the analyzed bacterial species in relation to the age group. The differences in the proportions of the bacterium in patients from the two age groups were calculated with the Mann–Whitney test.

## 3. Results

The clinical laboratory analysis indicated that *A. actinomycetemcomitans* was detected in 27% of the dental plaque samples from the individuals and the same proportion (27%) was positive for *P. gingivalis*. Conversely, *P. micra* was about twice as prevalent (49%) compared to the other two species (Table 1A). We further investigated the distribution of those three species according to age categorization in younger (≤35 years) or older patients (>35 years). Perhaps not unexpectedly, the prevalence of *A. actinomycetemcomitans* was found to be more than twice as high (OR = 2.84, 95% CI; 2.23–3.64) in individuals from the younger than the older group (*p* < 0.001) (Table 1A). The prevalence of *P. micra* was significantly lower in individuals from the younger age group (OR = 0.50, 95% CI; 0.40–0.63) (*p* < 0.001), whereas *P. gingivalis* was slightly lower distributed (OR = 0.74, 95% CI; 0.59–1.00) in the younger age group (*p* = 0.047) (Table 1A). When the species’ distribution was analyzed according to sample number, the prevalence distribution followed a similar trend as the patient-based distribution (Table 1B). The prevalence of *A. actinomycetemcomitans* was found to be more than twice as high (OR = 2.96, 95% CI; 2.50–3.50) in samples from the younger than the older group (*p* < 0.001) (Table 1A). The prevalence of *P. micra* was significantly lower in samples from the younger age group (OR = 0.43, 95% CI; 0.37–0.50) (*p* < 0.001), whereas *P. gingivalis* was similarly distributed (OR = 0.78, 95% CI; 0.78–0.93) in the two age groups (*p* = 0.006) (Table 1B).

The distribution of the three species among younger and older patients was also stratified according to their proportions among the total bacterial population in the samples (Table 2). Higher subgingival proportions of *A. actinomycetemcomitans* (0.1–25%) were observed in the older group, whereas this species was more dominant in proportions >25% in the younger group (*p* < 0.001). When the two other bacterial species were considered individually, the proportions of *P. gingivalis* were similarly distributed in the two age groups (*p* = 0.547), while *P. micra* were more dominant in the older age group (*p* = 0.001) (Table 2). *P. gingivalis* represented >25% of the total bacterial community in >60% of the plaque samples, irrespective of the age group. On the contrary, *P. micra* represented <25% of the total bacterial community in >80% of the plaque samples, irrespective of the age group (Table 2).

**Figure 1:**
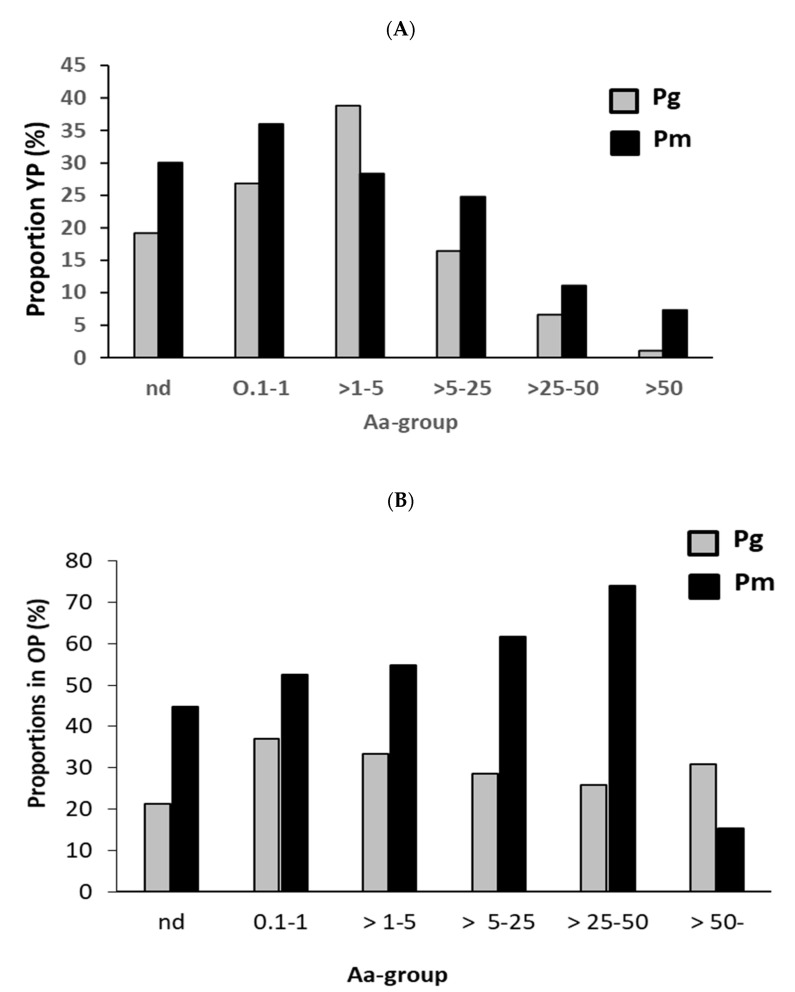
Distribution of *P. gingivalis* and *P. micra* in subgingival plaque samples, according to various proportions of *A. actinomycetemcomitans* present in the samples collected from young patients (YP) (**A**) or old patients (OP) (**B**).

Due to the difference in the proportion dynamics between *P. gingivalis* and *P. micra*, their co-existence was further investigated according to the distribution of *A. actinomycetemcomitans* in the samples (Figure 1). Subgingival plaque samples from younger patients (YP) displayed a gradual increase in *P. gingivalis* and *P. micra* with increasing *A. actinomycetemcomitans*, to 1 and 5% of the total bacterial load, respectively (Figure 1A). When *A. actinomycetemcomitans* exceeded 5% of the total bacterial load, both bacterial species displayed a decrease in proportions. On the contrary, in the older patient group (OP), the proportions of *P. gingivalis* remained unchanged, with increasing *A. actinomycetemcomitans* proportions. Instead, *P. micra* proportions co-increased with ascending proportions of *A. actinomycetmcomitans* but declined when the latter exceeded 50% (Figure 1B).

**Figure 2:**
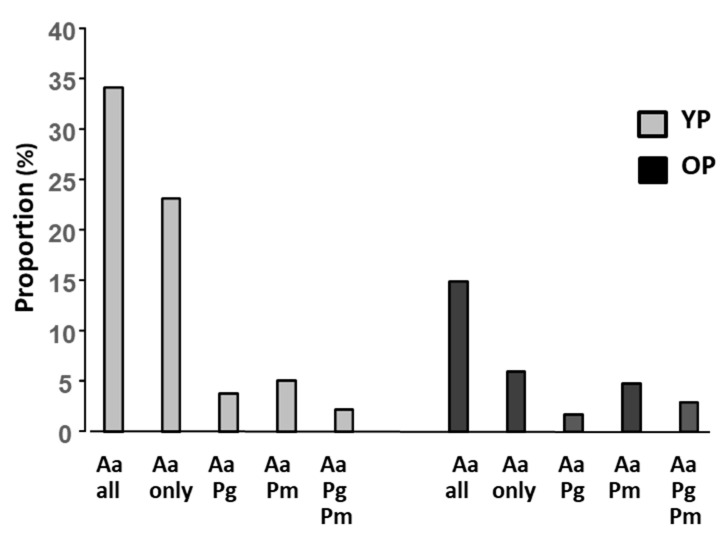
Prevalence of *A. actinomycetemcomitans, P. gingivalis*, and *P. micra* (n; %) in samples from young (YP) (grey) and old (OP) (black) patients, alone or in various combinations.

In a polymicrobial infection site the bacterial species may affect each other. To obtain an overview of co-colonization, the presence of the bacterial species was presented alone and in different combination (Figure 2). Among the samples from YP, the proportion of samples containing only *A. actinomycetemcomitans* was around two times higher than the samples containing *A. actinomycetemcomitans* in combination with the other bacterial species. Among the OP samples, the opposite was observed, i.e., the proportion of samples containing only *A. actinomycetemcomitans* was about two times lower than the samples containing *A. actinomycetemcomitans* in combination with the other bacterial species. Furthermore, it was noted that the proportion OP samples containing *A. actinomycetemcomitans* in combination with *P. micra* was almost three times higher than in samples when *P. micra* was replaced by *P. gingivalis*.

**Figure 3:**
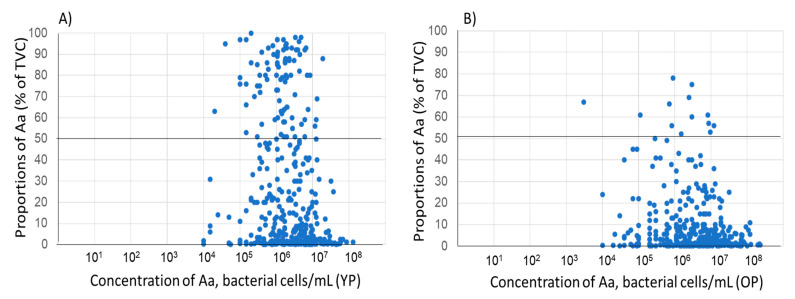
Proportion of *A. actinomycetemcomitans* in relation to concentration of the bacterium in the corresponding sample (cells/mL, log 10). (n = 723). (**A**) Young patients (YP) (n = 371). (**B**) Old patients (OP) (n = 352).

When the proportion and concentration of *A. actinomycetemcomitans* in the individual samples were compared, no overall relation could be observed, neither for the samples collected from young patients nor from old patients. (Figure 3A,B). However, in YP, the number of samples with a high proportion (>50%) was higher compared with that in the samples from the OP.

## 4. Discussion

The present study examined the dynamics of three well-established periodontal pathogens in the subgingival dental plaque of younger and older individuals. Aging is indeed a physiological process and is accompanied by natural changes in the composition of the oral microbiota [21]. In the present study, a higher prevalence of *A. actinomycetemcomitans* is shown in younger rather than older adults, whereas the reverse was the case for *P. micra*, and there was no differences in the prevalence of *P. gingivalis* between age groups. Our findings converge with earlier findings demonstrating that the prevalence of *A. actinomycetemcomitans* decreases with age but diverge in that the prevalence of *P. gingivalis* by increases with age [26,27,28]. When looking at the proportions of *P. gingivalis* in dental plaque, we did not find variations between the two age groups. Of note, in a cohort of, on average, 68-year-old patients, *P. gingivalis* has been subgingivally detected with higher frequency in periodontitis than health, but with no statistically significant differences in the bacterial counts [11].

In the case of *A. actinomycetemcomitans*, the current and earlier findings clearly agree that it is found in greater proportions in the subgingival dental plaque of younger individuals [11,23,26,27]. In an adolescent population in Ghana, the JP2 genotype of *A. actinomycetemcomitans* showed higher concentrations in subgingival samples compared with other genotypes of this bacterium [29]. In addition, salivary samples from Moroccan adolescents showed significantly increased concentrations of the JP2 genotype in individuals with periodontal attachment loss compared with that in saliva from periodontally healthy adolescents [30].

The case of *P. micra* is somehow less studied in the literature. We hereby show that *P. micra* is twice as prevent in subgingival plaque in the older compared to the younger population, whereas the proportions of this species in subgingival plaque do not vary according to the age grouping. While we have not identified studies reporting the subgingival prevalence of *P. micra* according to age grouping, there is evidence that the subgingival proportion of this species does not vary with age, but rather with periodontal heath status [20,31]. Of interest, in a cohort of, on average, 67-year-old patients, *P*. *micra* was subgingivally detected at higher numbers in active compared to stable periodontitis [32,33]. Similar results were noticed for the periodontitis-associated bacterial species *Tannerella forythus* and *Prevotella intermedius* [32].

In this study we also observed altered dynamics between the proportions of *A. actinomycetemcomitans* and *P. gingivalis* or *P. micra* in subgingival plaque. The gradual decrease in the proportions of the latter two species with increasing proportions of *A. actinomycetemcomitans* in the younger patient group converges with microbial clustering observations, implying that inherent factors promote the coexistence of *P. gingivalis* with *P. micra*, but not with *A. actinomycetemcomitans* [34]. Hydrogen sulfide produced by both *P. micra* and *P. gingivalis* may be a such factor, on the biochemical level [35]. Another factor which may have impact on the relationship between the bacterial species is *P. micra*-induced activation of gingipains [17]. Furthermore, experimental studies using biofilms have also shown a competitive interaction between *A. actinomycetemcomitans* and *P. gingivalis* [36,37]. However, in the older population our findings show that the presence of *A. actinomycetemcomitans* does not affect the proportions of *P. gingivalis*, but there is a positive interrelationship with *P. micra* instead.

The introduction of PCR-based quantification of periodontitis-associated bacterial species has in many aspects facilitated the work. However, quantification of the total amount of bacteria using PCR-based methods encounters problem [38], which in turn results in problems when determining proportions of the present bacterial species. In addition, normalization of the results requires determination of the sample size. Methods for this are scarcely reported. However, a method described by Thorbert Mros and co-workers [39] is based on usage of a curette corresponding to 1 μL (1 μL inoculation loop; Sarstedt).

In this study it is shown that there is no or a weak association between proportion and concentration of *A. actiniomycetemcomitans* in the samples. This further indicates that a high proportion of the bacterium in a sample does not necessarily mean that the sample contains a high concentration of the bacterium, or vice versa.

Clinical laboratory diagnostics is today recommended as the relevant treatment for aggressive or refractory forms of periodontitis. In addition, to analyze the samples for presence/proportions of periodontitis-associated bacterial species, it would be beneficial to screen the samples for the presence of JP2- and *cage E* carriers of *A. actinomycetemcomitans* [15,40]. Carriers of the JP2 genotype have shown a significantly increased risk of developing periodontal attachment loss compared to adolescents without this bacterium [13,14,41].

Based on the findings in this study of *A. actinomycetemcomitans*, *P. gingivalis* and *P. micra*, alone and in combination with each other among young and old patients, this could be inferred to ecological interdependencies between these species in the subgingival milieu, under the factor of aging. However, there is no concrete biological evidence at present to support this hypothesis.

A limitation of this study is the lack of clinical parameters; however, a sample from a periodontal pocket sent for microbial analysis is indicative for a diagnosis of periodontitis.

## 5. Conclusions

Periodontal plaque samples from young patients showed an increased presence and proportion of *A. actinomycetemcomutans* compared with samples from old patients, while *P. gingivalis* was similarly distributed in the two age groups. *P. micra* showed an increased presence and proportion in samples from old patients compared with the young patients.

## Figures and Tables

**Table 1:** Prevalence of of *A. actinomycetemcomitans* (*Aa*)*, P. gingivalis* (*Pg*), and *P. micra* (*Pm*), (n; %) in subgingival plaque samples based on number of patients (**A**) and samples (**B**) (YP: younger patients <35 years. OP: older patients >35 years).

(A)					
Age	n	*Aa*, n (%)	*Pg*, n (%)	*Pm*, n (%)	*negative*
YP	424	179 (42.2)	99 (23.3)	156 (36.8)	122 (28.8)
OP	1081	221 (20.4)	307 (28.4)	582 (53.8)	286 (26.4)
All patients	1505	400 (26.6)	406 (27.0)	738 (49.0)	408 (27.1)
**(B)**					
Age	n	*Aa*, n (%)	*Pg*, n (%)	*Pm*, n (%)	*negative*
YP	1087	371 (34.1)	203 (18.7)	295 (27.1)	426 (39.2)
OP	2360	352 (14.9)	539 (22.8)	1095 (46.4)	907 (38.4)
All patients	3447	723 (21.0)	742 (22.1)	1390 (40.3)	1333 (38.7)

**Table 2:** Distribution of *A. actinomycetemcomitans*, *P. gingivalis*, and *P. micra* in subgingival plaque samples according to their proportions (%) of the TVC. Both age group (young patients ≤35 years; YP and older patients; OP) and total patient (YP + OP) distributions are presented.

	≥0.1–1%	>1–5%	>5–25%	>25–50%	>50%	
*Aa*						
YP	78 (21.0)	66 (17.8)	87 (23.5)	45 (12.1)	95 (25.6)	371
OP	116 (33.0)	84 (23.9)	112 (31.8)	27 (7.6)	13 (3.7)	352
YP + OP	194 (26.9)	150 (20.7)	199 (27.5)	72 (10.0)	108 (14.9)	723
*Pg*						
YP	9 (4.4)	26 (12,8)	49 (24.1)	44 (21.7)	75 (36.9)	203
OP	16 (3.03)	59 (10.9)	142 (26.3)	116 (21.5)	206 (38.2)	539
YP + OP	25 (3.4)	85 (11.4)	191 (25.7)	160 (21.6)	281 (37.9)	742
*Pm*						
YP	37 (12.5)	102 (34.6)	106 (35.9)	37(12.5)	13 (4.4)	295
OP	91 (8.3)	297 (27.1)	495 (45.2)	150 (13.7)	62 (5.7)	1095
YP + OP	128 (9.2)	399 (28.7)	601 (43.2)	187 (13.5)	75 (5.4)	1390

## Data Availability

Data are available from the corresponding author (R.C.).
